# Temporomandibular joint anchorage surgery: a 5-year follow-up study

**DOI:** 10.1038/s41598-019-54592-2

**Published:** 2019-12-13

**Authors:** Xiuming Liu, Zhihang Zhou, Yi Mao, Xuzhuo Chen, Jisi Zheng, Chi Yang, Shanyong Zhang, Liang Huo

**Affiliations:** Department of Oral Surgery, Ninth People’s Hospital, College of Stomatology, Shanghai JiaoTong University School of Medicine, Shanghai Key Laboratory of Stomatology & Shanghai Research Institute of Stomatology, National Clinical Research Center of Stomatology, Shanghai, China

**Keywords:** Dental diseases, Oral diseases

## Abstract

The purpose of this study was to confirm the 5-year efficiency of temporomandibular joint (TMJ) anchorage, using clinical evaluation and magnetic resonance imaging (MRI). We also studied the influence of disc length and position on efficiency and postoperative condylar height. Sixty-one patients (76 joints) undergoing TMJ disc anchorage were followed up for >5 years. Visual analogue scale (VAS) score and maximum mouth-opening pre-and postsurgery were analysed and patient satisfaction recorded. Disc length, condyle height and disc position pre- and postsurgery were measured using MRI. Patients were ranked as A, B or C degree according to postoperative disc condyle position. Mean follow-up time was 71.34 months. Maximum mouth-opening improved by 14.34 ± 5.87 mm, and VAS score decreased by 33.44 ± 20.56 (P < 0.05). Clinical evaluation efficiency was 84.21%; patient satisfaction rate was 85.53%. On follow-up MRI, 68 joints were judged A or B degree (89.67%). Disc length was 7.96 ± 1.38 mm, 7.10 ± 1.41 mm and 5.75 ± 1.16 mm in A, B and C groups, respectively. In patients evaluated as C, condylar height decreased by 0.43 ± 1.36 mm, while increasing by 0.67 ± 1.88 mm and 0.51 ± 1.09 mm in A and B groups, respectively (all P < 0.05). We concluded that anchorage surgery improves mouth-opening and eliminates pain, longer disc length is related to better postoperative disc position, and significant condylar reconstruction occurs after disc repositioning. MRI confirmed that TMJ disc anchorage is reliable 5 years postsurgery.

## Introduction

Disc repositioning was accepted as an optional surgical technique after Wilkes described the function of the temporomandibular joint (TMJ) according to form and anatomy, using arthrography^1^. Previous reports show variable results from disc repositioning procedures. Lack of long-term stability is the main cause of failure. Wolford^2^ solved this problem by developing a TMJ anchorage procedure, which uses a bone anchor to reposition the protruding disc in TMJ dysfunction. One study following up the jaw function and maximum incisal opening of 102 patients (188 sides) for a mean of 46.2 months (range 14–84 months) using visual analogue scales (VASs) demonstrated satisfying clinical results^2^. To improve success rate, reposition stability and implant safety, Yang *et al*.^3^ applied a Chinese-made anchor nail in a modified disc anchor surgery.

Following the development of TMJ anchorage, magnetic resonance imaging (MRI) has emerged as the most important method for both preoperative and postoperative evaluation of TMJ dysfunction. According to the classical description, the posterior band of the disc is located above the condylar head when the mandibular condyle is centrically positioned in the fossa^4,5^. Surgeons^3^ made an evaluation of 81 joints immediately after modified TMJ disc repositioning with a Chinese-made anchor nail, using MRI, with a total of 71 joints (87.7%), which showed excellent results. However, MRI has not been used in other long-term follow-up studies.

Our study used both clinical and MRI evaluation for cases 5 years after anchorage surgery. VAS score and maximum mouth-opening measurement were recorded as the criteria of clinical efficiency. Patient satisfaction was also evaluated. To evaluate effect, disc position before and after surgery were analysed using MRI. We also studied the influence of disc length on disc position and the effect of disc position on postoperative condylar reconstruction. Finally, we compared the results of the three evaluation methods (clinical evaluation, patient satisfaction and MRI) to confirm the consistency of these methods.

## Results

Mean preoperative VAS value was 43.93 ± 21.85 (range: 0–80), and 10.49 ± 19.18 (0–60) postoperatively, an improvement of 33.44 ± 20.56 (paired t test: t = 11.48, P < 0.0001).

Mean disc length for class A was 7.96 ± 1.38 mm and for class B 7.10 ± 1.41 mm (unpaired t test: t = 0.2169, P = 0.8289). A comparison between class A and C as well as between B and C also demonstrated significant differences in mean disc length (unpaired t-test: t = 5.110, P < 0.0001 and t = 3.616, P = 0.0013, respectively), revealing that postoperative disc condyle position is related to disc length.

**Figure 1:**
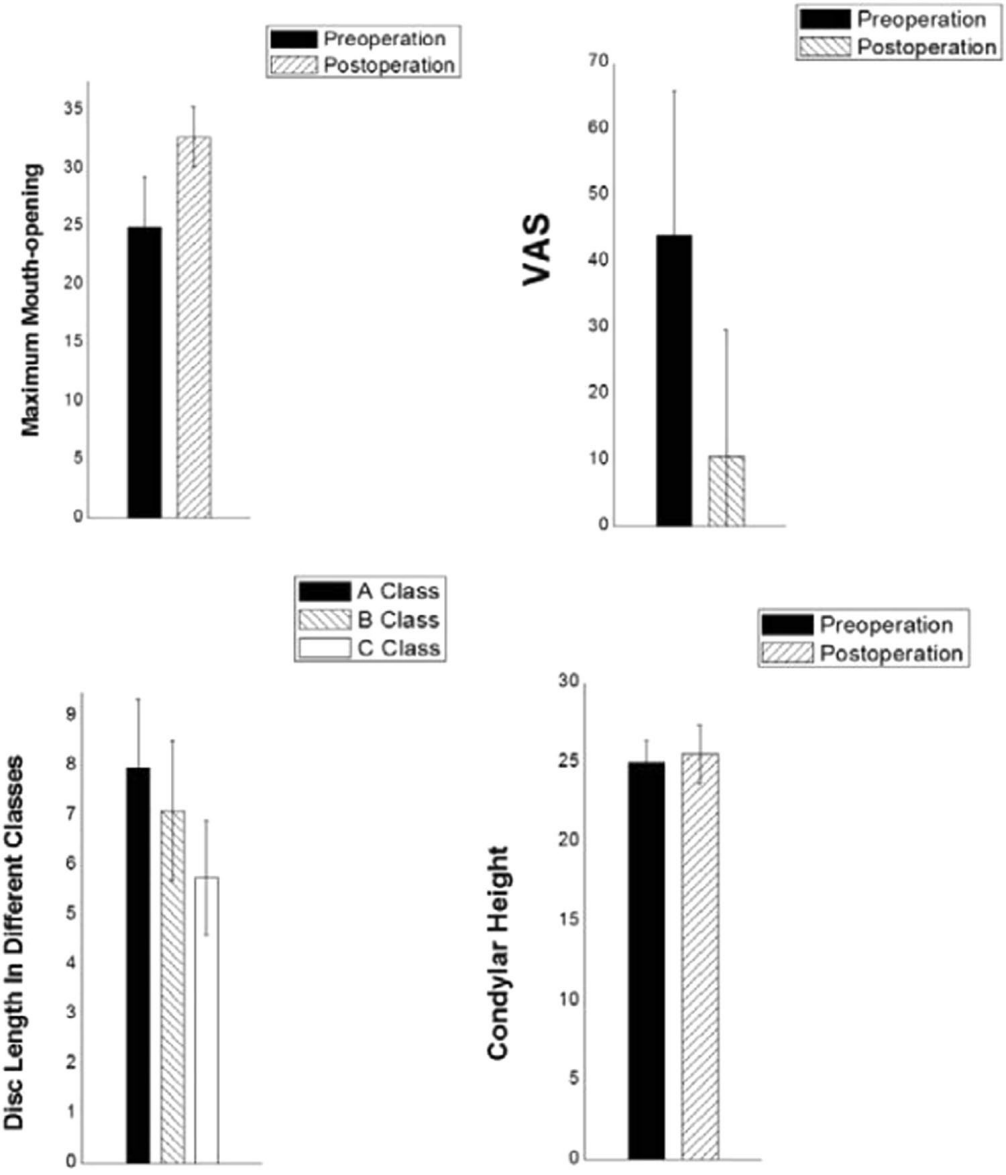
Maximum mouth-opening: 24.98 ± 4.29 mm (range 15–32 mm). Postoperative mouth-opening 32.67 ± 2.57 mm (25–39 mm), an improvement of 7.69 ± 5.23 mm. T-test revealed statistical significance (t = 11.48, P < 0.01).

Mean preoperative condylar height was 25.01 ± 1.37 mm (21.84–30.19 mm) and postoperative height was 25.66 ± 1.84 mm (21.92–30.59 mm), a statistically significant difference (P = 0.0274; Fig. 1).

In patient self evaluation, 26 joints (34.21% [26/76]) were rated by patients as satisfactory, 40 joints (52.63% [40/76]) were rated as basically satisfactory, and the remaining 11 joints 13.16% (10/76) were rated as unsatisfactory. Overall patient satisfaction was 86.84% (66/76).

**Figure 2:**
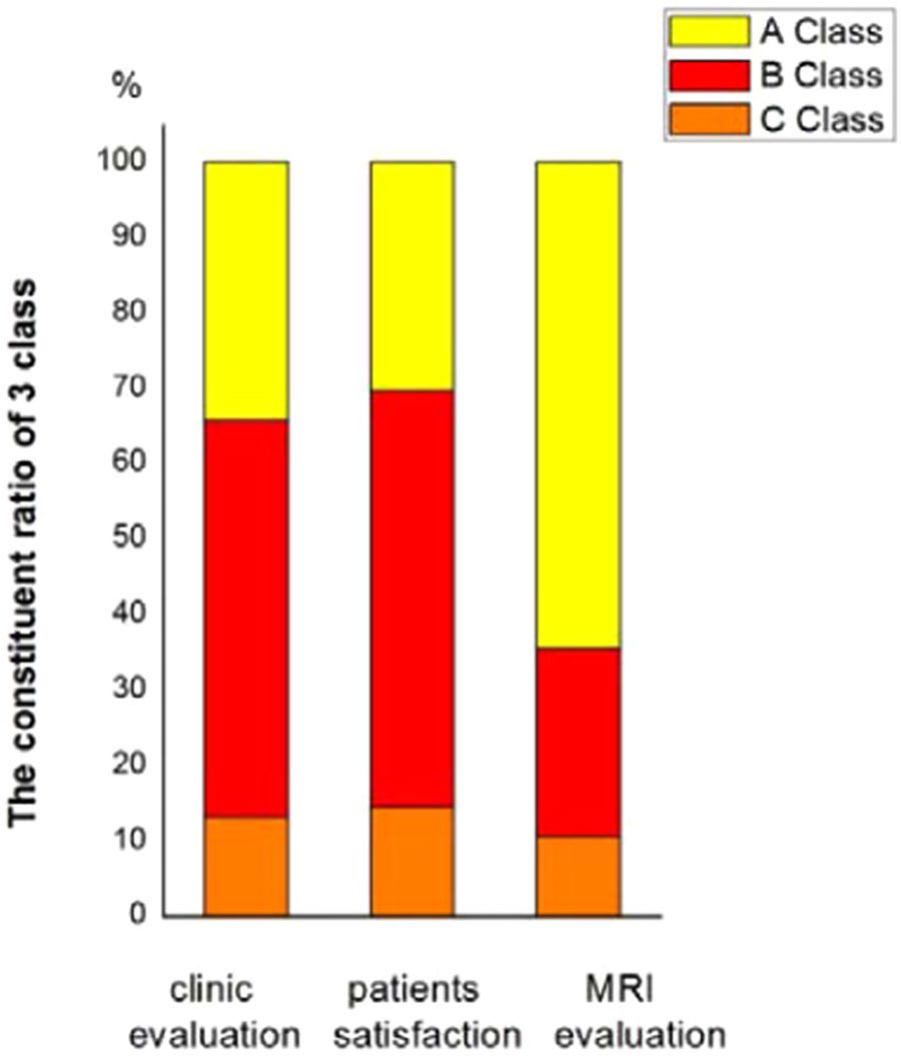
Of 61 cases (76 sides) of anchorage surgery, the curative effect for group A was 30.26% (23/76), the curative effect for group B was 55.26% (42/76) and the curative effect for group C, 14.47% (11/76), giving a total curative effect of 85.52% (65/76).

According to MRI, 49 joints were classified as A, while 19 joints were classified as B, where both classes represented successful cases. These figures indicate no statistically significant difference in the efficiency of the different evaluation methods (P > 0.7584; Fig. 2).

## Discussion

Clinical evaluation of TMJ treatment may include assessment of joint clicking and limitations to mouth-opening caused by internal derangement (ID). However, there is no uniform standard for postoperative evaluation^6^. Some scholars regard mouth-opening as a success criterion, while others believe that multiple elements, including pain, should be considered. Even in the evaluation of mouth-opening, standards and methods vary, with some researchers using improved percentage of mouth-opening to record the curative effect. One study^7^ has reported 74% of patients as having complete remission in postoperative follow-up, although 83.8% of patients underwent orthognathic surgery simultaneously and thus the improvements may not be entirely due to TMJ anchorage. The researchers^8^ merely reported on theTMJ anchorage and did not mention postoperative evaluation. Results of a 20-year postoperative questionnaire of Abramowicz *et al*.^9^ demonstrated that 77% of patients who had received TMJ anchorage experienced relief from pain. MRI evaluation as part of long-term follow-up was not performed in any of the studies discussed above.

To ensure comparability and consistency, three different methods of evaluation of efficacy were compared, to ensure comparability and consistency? in our study group.

### Clinical evaluation

According to our clinical experience, the purpose of surgical treatment is to ease pain and/or improve maximum mouth-opening. We used VAS score and maximum mouth-opening as the clinical evaluation criteria. Mean increase in mouth-opening was 7.69 ± 5.23 mm and mean decrease in VAS score was 33.44 ± 20.56. Patients with anterior TMJ disc displacement without reduction were included, and 56.58% (43/76) of the joints were stage III or above. Change in maximum mouth-opening was obvious and significant (t = 11.48, P < 0.01), clinically confirming the 5-year effect of the anchorage procedure.

### MRI evaluation criteria

MRI is the ideal imaging method for evaluating TMJ dysfunction. Firstly, bone structure and soft tissue, including the articular disc and the retrodiscal tissue, are clearly displayed. Secondly, multi-layer and multi-angle projection can be used without changing the posture of patients, comprehensively displaying the construction of organs and tissues. Thirdly, MRI is noninvasive and nonradioactive, an obvious superiority over other imaging examinations. Despite being the optimal and most effective diagnostic method for TMJ dysfunction, MRI has not been used in long-term follow-up studies to evaluate the precise long-term effects of operative condylar reconstruction. We performed MRI on patients 5 years after surgery to explore the relationship between disc position and disc length and the reconstruction of condylar height in different groups.

To perform an accurate evaluation, the 12-o’clock condylar position must be located precisely. The reference plane and centre contact methods were used to locate the long axis of the condyle and the centre of the condylar head by previous scholars^5,10^, a technique with several limitations such as difficulty point-locating and applicability of different crowd. Referring to Nebbe’s three circle method^5,11,12^, we applied a two-circle method to locate the gauge points, which avoids the influence of the lower jaw’s spatial position and includes the angle between the condylar head and neck. In addition, with the displacement of the articular disc, the disc, including the posterior region of the disc, will be degenerated. During jaw movement, an abnormal disc–condyle relationship following displacement of the disc produces pathological pressure, which may cause folding and adhesion in the thick area of disc. Because of decreased internal hydration, the disc shrinks^13^ and cannot totally cover the condyle after repositioning. Based on this knowledge, the evaluation of TMJ treatment using routine methods would result in bias. There is little literature available on the MRI evaluation of TMJ disc repositioning, thus no accepted and established criteria are available about the positional relationship of the TMJ disc after disc repositioning. We used our own standards as follows: the posterior margin of posterior band should be after the 12-o’clock point, with an ideal position between 12 and 1 o’clock. Three layers (lateral, central and medial) in the sagittal plane were selected to obtain accurate estimation. TMJ disc was marked as A if it was completely set in all three layers, or as B if in two layers or as C if in only one layer. Our criteria were based on extensive clinical experience and knowledge of relapse prevention. Moreover, we observed posterior and anterior changes at the sagittal plane and provided objective imaging based on the accurate repositioning of the displaced disc, according to the evaluation criteria stated above. Abiding by this role, the disc should shield the condyle from excessive load, even if it does not fully cover the front bevel. Instead of anatomical repositioning, anchorage requires functional repositioning. For now, these evaluation criteria provide objective imaging evidence and are considered a scientific and rational evaluation method.

The results of this study show that MRI is an effective method for evaluating the disc–condylar relationship, with high rate of anchorage after 5 years (91.89%). Postoperative stability was relative to disc length and this should be considered before the procedure. In summary, anchorage provides a reliable long-term effect.

### The effect of disc position on condylar bone reconstruction

**Figure 3:**
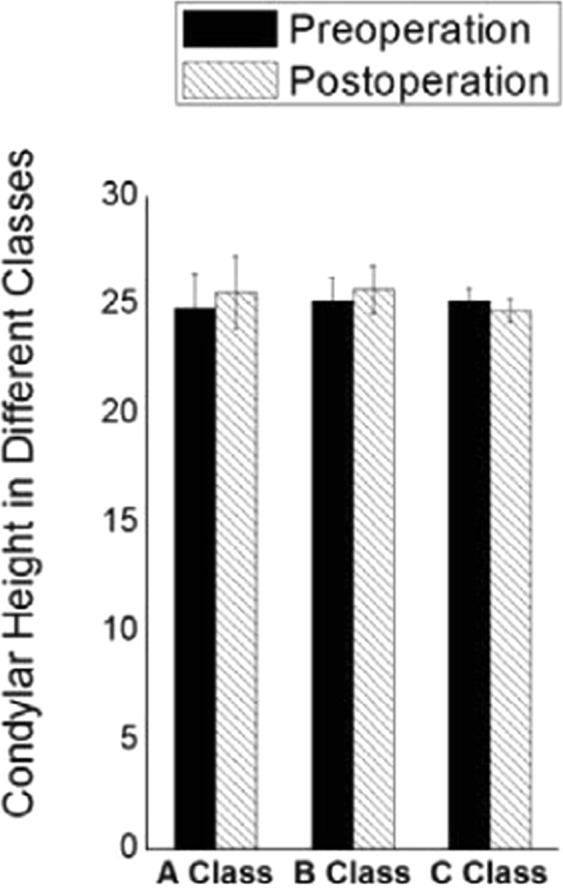
In comparison of pre- and postoperative disc–condylar position, condylar height change was significant for group A (P = 0.0411), but not for group B (P = 0.1488) or group C (P = 0.3331).

Normally, the condyle remodels under pressure from different directions during movement of the mandible and secondary movements. Following functional incapacitation of the disc after ID, progressive remodelling becomes degenerative remodelling^14,15^. The cartilage of the anterior slope of the condyle is affected, which, in modern conception, is a biological unit including the cartilage and the bone under it. This conception explains the progress of subchondral bone destruction: loaded pressure → subchondral tiny bone crack → change in cell factors → activation of related signalling pathways → cell activation → increased matrix secretion and destruction of tissue. Therefore abnormal stress must be interrupted before total loss of the progressive remodelling function, and condylar height can then be recovered. To confirm this, we analysed the relationship between disc position and change of condyle height (Fig. 3).

## Conclusion

Clearly it would be better to reposition the TMJ disc than reconstruct it. Postoperative disc position is relative to disc length according to our previous description, leading to the inference that disc length is essential to condyle reconstruction. In summary, an open procedure should be performed as early as possible.

## Materials and Methods

### Patients

This was a longitudinal retrospective study that was approved by our institutional review board. We followed the tenets of the Declaration of Helsinki for research involving human subjects, and all participants signed an informed consent agreement (ID: 2017422T318).

The included patients were recruited from a consecutive series of patients referred to the TMJ division of the Department of Oral and Maxillofacial Surgery at the Shanghai Ninth People’s Hospital affiliated to Shanghai Jiao Tong University School of Medicine. The cohort comprised 61 patients (76 joints) who underwent modified TMJ disc repositioning in the hospital between January 2004 and February 2008 – 7 males and 54 females with a mean age of 39 ± 12.76 years (range: 14–64 years). The mean duration of follow-up was 71.34 months (60–112 months). In accordance with the diagnostic criteria of the Wilkes-Bronstein classification for TMJ disorders^1^, 34 joints were diagnosed as ID stage III, 36 as stage IV and 6 as stage V.

### Inclusion criteria

The following inclusion criteria were used:

1. Patients assessed as class A or B in immediate postoperative MRI evaluation.
2. Patient with complete clinical and MRI records for more than 5 years.

### Surgical technique

A standard endaural preauricular incision with an anterior release was made through the skin and a flap developed within the subcutaneous fat superficial to the temporoparietal fascia. The flap was elevated on the surface of the zygomatic arch to show the joint capsule. After completion of the anterior release, a periosteal elevator was wedged behind the condyle over the bilaminar tissue to evaluate for passive reduction of the disc. One self-drilling miniscrew with a slot in the tail was implanted in the posterior midcondyle, inferior to the posterior condylar slope. Two 3-0 nonresorbable polyester sutures were secured to the anchor. Two horizontal mattress sutures were placed at the junction of the disc and the retrodiscal tissue. One suture was placed through the medial aspect of the posterior band and another through its lateral aspect. A stabilising splint was used after surgery.

### Evaluation methods

Mobility was judged by the distance of vertical mouth-opening between the central incisors. The VAS comprised a 100-mm ruler, with the point on the ruler indicated by the patient. Zero millimetres represented complete absence of pain and 100 mm represented severe pain.

### Clinical criteria

Clinical standards were described as follows:

I. pain-free, central incisor vertical tooth space ≥35 mm and ability to eat, talk, etc. without hindrance.
II. relatively pain-free, central incisor vertical tooth space ≥35 mm and the ability to perform oral functions with tolerable mild discomfort.
III. painful, central incisor vertical tooth space <35 mm and the ability to perform oral functions with tolerable discomfort.

Corresponding to I, II and II, self efficacy is divided into satisfied, generally satisfied and dissatisfied.

### MRI image evaluation standards

Complying with routine sequencing, an MRI scan was obtained using a 1.5-T imager (Signa, General Electric, Milwaukee, WI) with bilateral 3-inch TMJ surface coil receivers. To find its long axis, the condyle was scanned in the sagittal plane in a closed-mouth position using a T1-weighted spin echo sequence (TR: 500 ms, TE: 25 ms, FOV: 2 cm, depth 1 mm, window width 256 × 192). The sagittal plane was determined to be perpendicular to the long axis, as the coronal plane paralleled the long axis. A T1-weighted spin echo sequence in the sagittal plane in the open-mouth and closed-mouth position, so as coronal plane. A T2-weighted spin echo sequence was scanned using the same routine (TR: 3000 ms, TE: 30 ms, FOV: 2 cm, depth 1 mm, window width 256 × 192). For the same patient, 1-mm thickness scans were performed before and after the operation. In the sagittal plane, the internal, middle and lateral layers of the long axis of the condylar process were compared. Anterior, middle and posterior layers were selected in the coronal plane to compare the position of the disc before and after surgery.

The largest cross section of disc was selected in the oblique sagittal plane. Point B was regarded as the narrowest area of the disc. Point A and Point C were selected as the two poles of the disc and the distance between Point A and Point C was the length of disc.

**Figure 4:**
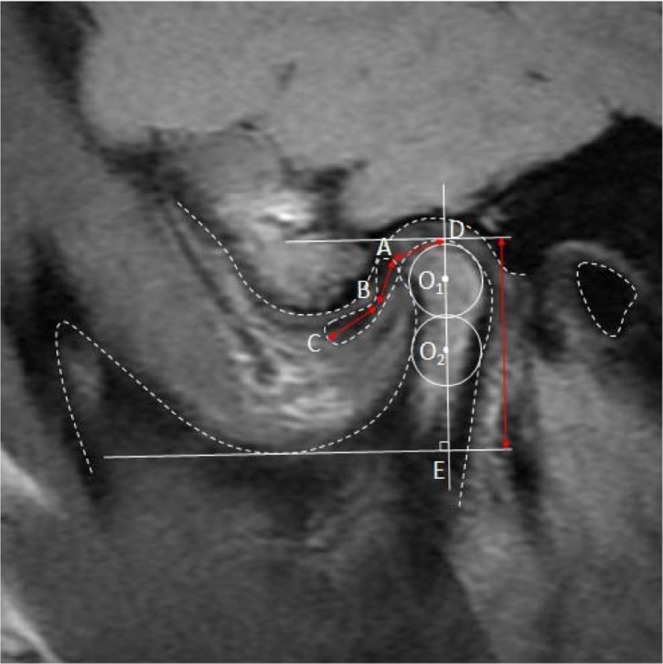
Key reference points on MRI measurement. Point A is located in the posterior border of the disc; point B is located in the centre of the disc; point C is located in the anterior border of disc; point D is the 12-o’clock position on the head of condyle; point E is the intersection of the condyle axis and its perpendicular cutting sigmoid notch. Disc length is the total of the length of line AB and line BC. Line DE shows the height of the condyle.

The long axes of the condylar head and neck were determined using an improved two-step method^6^. First, the largest circle internally tangent to the outline of the anterior, posterior and superior surfaces of the condylar head was drawn, and labelled O1. Then an internally tangent circle, O2, was drawn at the most curved area between the condylar head and neck. Next, the long axis of the condyle was defined as the line passing through the centres of circles O1 and O2. A line perpendicular to the long axis of the condyle and tangent to the contour of the mandibular notch was drawn as a horizontal reference line. The translational horizontal reference line was tangent to the top of the condyle, and the tangent point D was the top point of the condyle. Regarding D as the 12-o’clock point, it would be estimated as re-set if the posterior margin of the posterior band was after the 12-o’clock point (Fig. 4). The ideal position was between 12 and 1 o’clock.

**Figure 5:**
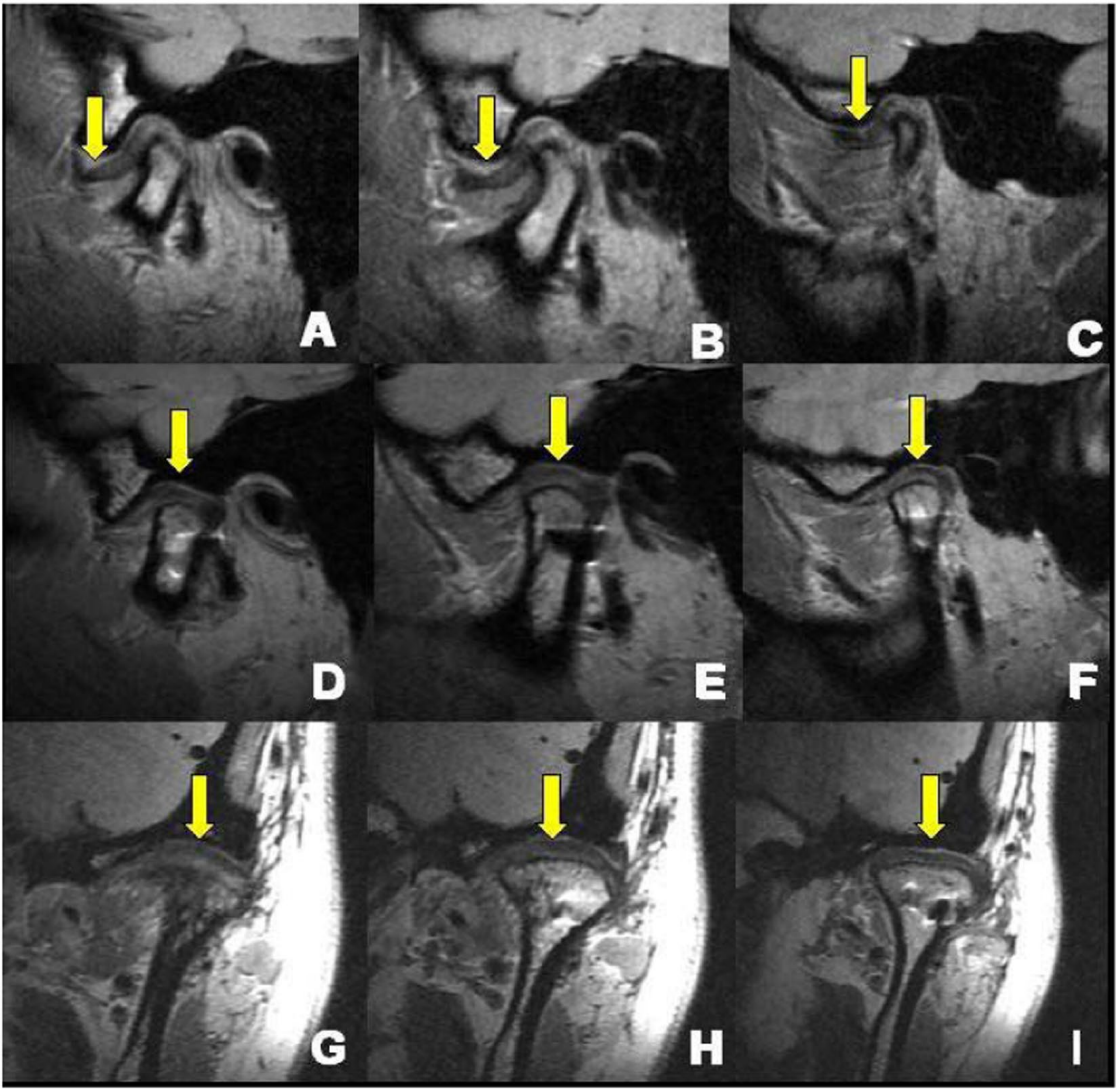
Disc position visualised by MRI before and after surgery – example of A classification. (**A**–**C**) show the displaced disc anterior to the condyle (yellow arrow). (**D**–**I**) show the displaced disc repositioned to the normal position in two different directions (yellow arrow). Based on the imaging findings this case was classified as (**A**).

**Figure 6:**
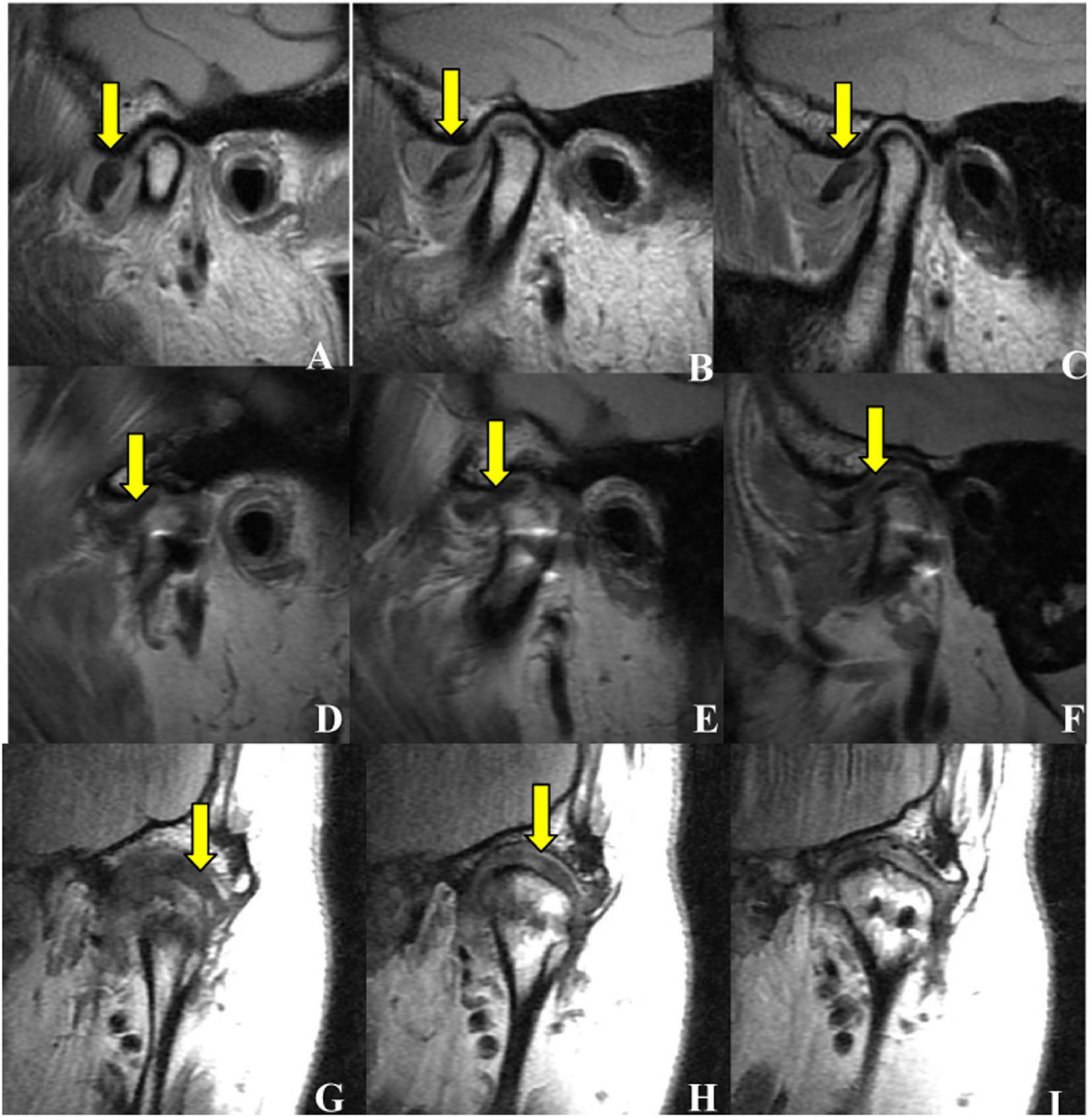
Disc position visualized by MRI before and after surgery – example of B classification. Internal (**A**), middle (**B**) and lateral (**C**) layers of the long axis of the condylar process shown before surgery, in the sagittal plane. The images demonstrate that the disc is anteriorly displaced in the closed-mouth position (yellow arrow). Internal (**D**) and middle (**E**) layers of the long axis of the condylar process after surgery, in the sagittal plane. The images demonstrate that the displaced disc is repositioned in the normal position (yellow arrow), while in the lateral (**F**) layer the displaced disc is not repositioned (yellow arrow). Anterior (**G**) and middle (**H**) layers of the long axis of the condylar process, after surgery, in the coronal plane. The images demonstrate that the displaced disc is repositioned in the normal position (yellow arrow), while in the posterior (**I**) layer the displaced disc is not found. As the disc is re-set in two layers (internal and middle layers in the sagittal plane, and anterior and middle layers in coronal plane) the case was classified B.

**Figure 7:**
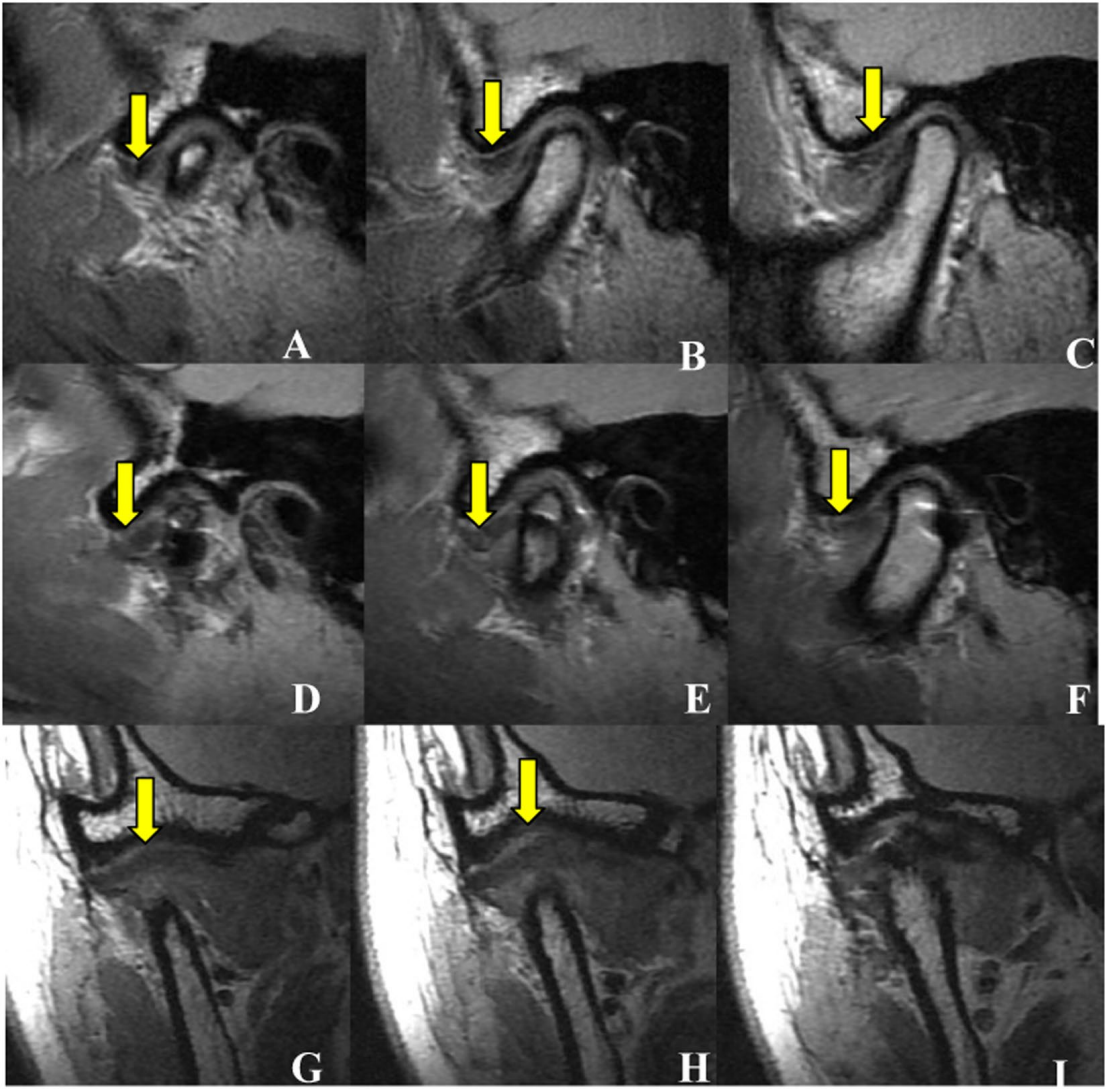
Disc position on MRI before and after operation – example of C classification. Internal (**A**), middle (**B**) and lateral (**C**) layers of the long axis of the condylar process shown before surgery, in sagittal plane. The images demonstrate that the disc is anteriorly displaced in the closed-mouth position (yellow arrow). Internal (**D**), middle (**E**) and lateral (**F**) layers of the long axis of the condylar process after operation, in sagittal plane. The images demonstrate that the displaced disc is not repositioned (yellow arrow). Anterior (**G**) and middle (**H**) layers of the long axis of the condylar process after surgery, in coronal plane. The images demonstrate that the displaced disc is not repositioned (yellow arrow), and in the posterior (**I**) layer the displaced disc is not found. As the disc is not re-set in any layers, the case was classified C.

**Figure 8:**
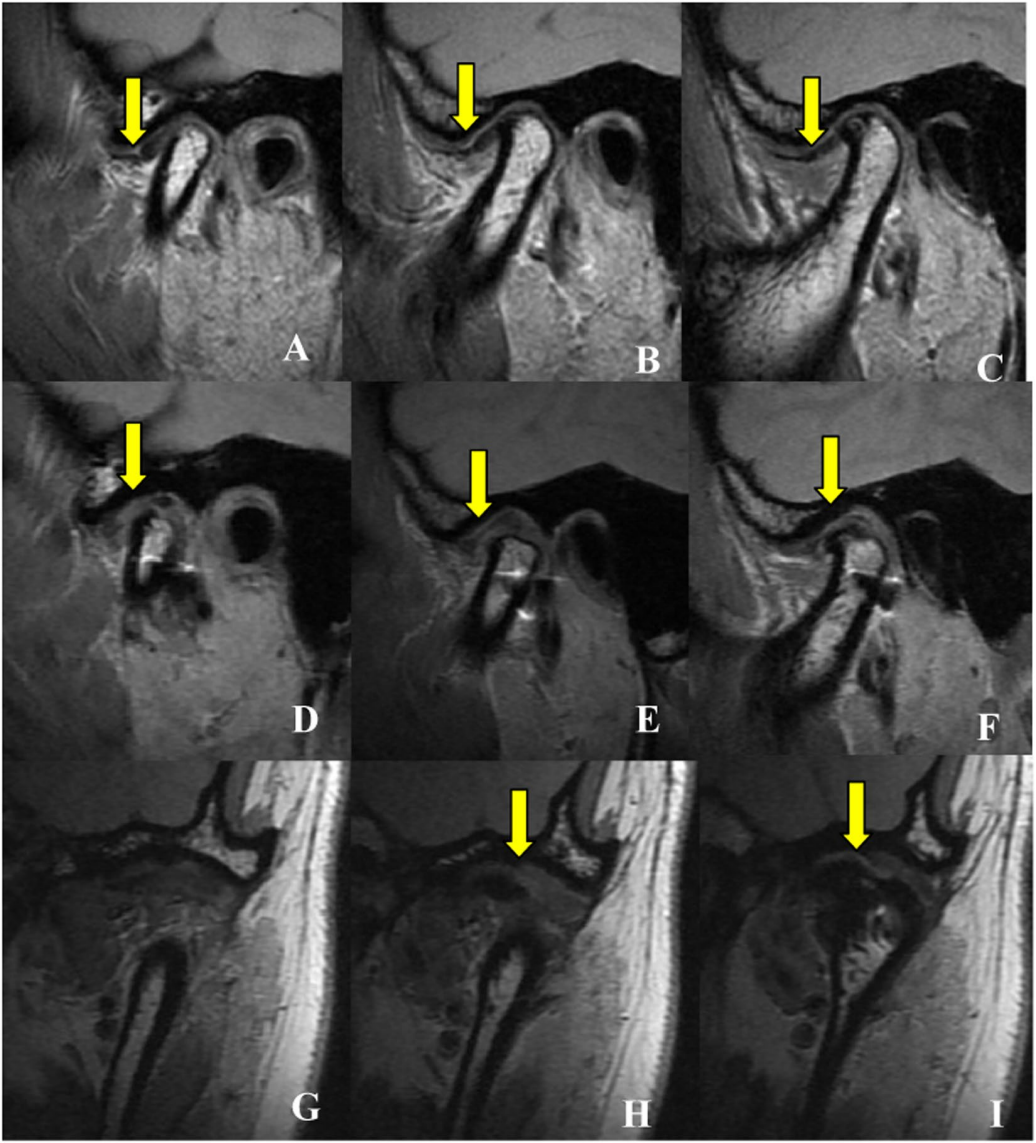
Female patient aged 26 years, with internal derangement stage IV right side and stage V left side, disc position on MRI before and after surgery to left side. Internal (**A**), middle (**B**) and lateral (**C**) layers of the long axis of the condylar process before surgery, in sagittal plane. Images demonstrate that the disc is anteriorly displaced in the closed-mouth position and the disc is thin with a potentially broken area (yellow arrow). Internal (**D**), middle (**E**) and lateral (**F**) layers of the long axis of the condylar process after surgery, in sagittal plane. Images demonstrate that the displaced disc is repositioned to the normal position but the disc image appears broken (yellow arrow). Middle (**H**) and posterior (**I**) layers of the long axis of the condylar process, after surgery, in coronal plane. The images demonstrate that the displaced disc is repositioned to the normal position but the disc image appears broken (yellow arrow), and in the anterior (**G**) layer the displaced disc is not seen.

Scans of 1-mm thickness were performed before and after surgery. In the sagittal plane, internal, middle and lateral layers of the long axis of the condylar process were compared. Anterior, middle and posterior layers was selected in the coronal plane to compare the position of the disc before and after surgery. To evaluate the effect of surgery the following classifications were used: A if a complete re-set in all layers is shown (Fig. 5), B if it is shown in two layers (Fig. 6) and as C if it is shown in less than one layer (Fig. 7). Figure 8 shows a broken disc. Each estimation was performed by two experienced doctors and adjudicated by a third doctor in cases of difference in judgement between the first two assessors.

### Statistical analysis

Differences in vertical mouth-opening distance, VAS scores and condylar height were evaluated using t-test. Mean preoperative disc length and mean age were analysed using chi-square test. Fisher’s test was chosen to estimate clinical effectiveness, patient satisfaction and efficiency of MRI. SAS software version 8.2 for Windows was used for the analyses and P values smaller than 0.05 were considered statistically significant.

### Ethics approval

The method and protocol complied with the Ethics Committee of the Shanghai Ninth People’s Hospital guidelines and all participants gave informed consent (ID: 2017422T318).
